# Increase in Synchronization of Autonomic Rhythms between Individuals When Listening to Music

**DOI:** 10.3389/fphys.2017.00785

**Published:** 2017-10-17

**Authors:** Nicolò F. Bernardi, Erwan Codrons, Rita di Leo, Matteo Vandoni, Filippo Cavallaro, Giuseppe Vita, Luciano Bernardi

**Affiliations:** ^1^International Laboratory for Brain, Music and Sound Research, Montréal, QC, Canada; ^2^Department of Psychology, McGill University, Montréal, QC, Canada; ^3^Department of Public Health and Neuroscience, University of Pavia, Pavia, Italy; ^4^Fondazione Ospedale San Camillo IRCCS, Venice, Italy; ^5^Neurology Unit, Department of Clinical and Experimental Medicine, University of Messina, Messina, Italy; ^6^Folkälsan Research Center, Folkälsan Institute of Genetics, University of Helsinki, Helsinki, Finland

**Keywords:** music, group synchronization, cardiovascular rhythms, music listening, respiratory rhythms, generalized partial directed coherence, physiological rhythms

## Abstract

In light of theories postulating a role for music in forming emotional and social bonds, here we investigated whether endogenous rhythms synchronize between multiple individuals when listening to music. Cardiovascular and respiratory recordings were taken from multiple individuals (musically trained or music-naïve) simultaneously, at rest and during a live concert comprising music excerpts with varying degrees of complexity of the acoustic envelope. Inter-individual synchronization of cardiorespiratory rhythms showed a subtle but reliable increase during passively listening to music compared to baseline. The low-level auditory features of the music were largely responsible for creating or disrupting such synchronism, explaining ~80% of its variance, over and beyond subjective musical preferences and previous musical training. Listening to simple rhythms and melodies, which largely dominate the choice of music during rituals and mass events, brings individuals together in terms of their physiological rhythms, which could explain why music is widely used to favor social bonds.

## Introduction

It has been argued that music may have been developed over the course of human evolution as a tool to create and strengthen social bonds amongst interacting group members (Kogan, 1997; Dunbar, 2012). Through which precise mechanisms such bonds would be generated is still largely unknown (Tarr et al., 2014). The embodied cognition framework has developed the idea that the human body would function as a mediator for meaning formation, such that feelings and concepts would be grounded in psychophysiological and sensorimotor networks (Leman and Maes, 2014). The autonomic nervous system constitutes a primary candidate in this respect, as visceral responses constitute one of the key substrates of the emotional experience (Scherer, 2005). By inducing similar physiological responses across individuals, music could provide a biological foundation for reciprocal understanding and rapport (Levenson and Ruef, 1997), something we refer to as the musical physiological bonding hypothesis.

The fact that music influences autonomic function similarly across individuals (Bernardi et al., 2006, 2009), for example by increasing or decreasing the average heart rate, provides support to what we refer to as a *weak version* of the musical physiological bonding hypothesis. In this study we searched for evidence of what we term the *strong version* of the musical physiological bonding hypothesis, namely that music will not simply influence the physiology of different individuals in a similar fashion on average, but that music will actually *synchronize* the continuous physiological rhythms among the listeners.

Here we collected physiological responses from several individuals simultaneously as they listened to a live organ concert in a naturalistic environment. We chose a live concert design because listening to live music has been shown to yield superior entrainment between music and body physiology, compared to recorded music (Shoda et al., 2016), which in turn is likely to maximize the chances of inter-individual synchronization to occur. We took advantage of the possibility of using one of the most versatile organs available in Europe, capable of imitating a large number of musical instruments. By means of recording four partly independent physiological endpoints such as, heart rate, breathing rate, peripheral circulation, and blood pressure, we evaluated the inter-individual synchronization of physiological rhythms while passively listening to music.

In consideration of the wide variety of music genres and styles, we also speculated that different musical excerpts could yield different degrees of interpersonal synchronization. In an exploratory analysis of this idea, we asked whether the degree of interpersonal synchronization could be explained by differences in subjective appreciation and/or by basic auditory features of the music. Based on previous studies showing that preferred music triggers stronger emotional and physiological responses (Blood and Zatorre, 2001; Brattico and Jacobsen, 2009), we hypothesized that music receiving higher scores of subjective appreciation may result in stronger group synchronization. Furthermore, based on recent findings showing that simpler rhythmic patterns prompt stronger group motor synchronization (Codrons et al., 2014), we hypothesized that music excerpts with simpler acoustic structure may result in greater interpersonal synchronization of physiological rhythms.

We did not aim at comparing the responses to music in social setting with the responses when listening alone (as, for example, in Sutherland et al., 2009). In principle, investigating autonomic synchronization between participants does not require the listeners to be physically together, as the coherence between individuals' time series could be reconstructed *post-hoc* from participants studied in isolation. On the other hand, the validity of such an approach would be questionable, and one would still have to show that a *post-hoc* synchronization holds when participants are brought together and exposed to a more naturalistic, and hence intrinsically noisier, situation.

Music training has been shown to yield profound changes in auditory brain structures and function, at both cortical and subcortical level (Kraus and Chandrasekaran, 2010). As a result, it is possible that synchronization may occur only, or significantly more, in individuals with previous training compared to untrained individuals. By separately testing a group of musicians and one of non-musicians, here we sought to clarify whether prior music training is needed for individuals to experience group synchronization, or whether such synchronization may be found regardless of previous musical background.

## Methods

### Participants

We recruited two groups of participants, one with (*n* = 14) and one without (*n* = 13) prior musical training (see Supplementary Table 1 for detailed participants' information). The participants of each group were present at the same time in the location of the experiment. All participants gave written informed consent to the study, which was approved by the Ethics committee of the University of Pavia.

### Music excerpts

Recordings were performed during an initial resting baseline and while listening to seven organ music excerpts performed live by a professional organist in the Basilica Cattedrale Santa Maria Assunta of Messina (Italy). The details of the music excerpts are provided in Table 1 and the audio files of the music excerpts recorded live during the experiment are provided as Supplementary Material. The choice for this kind of music was based on the following two reasons. First, various religious traditions made a large use of organ music to convey spiritual messages beyond words. Thus, while certainly being specific to a certain listening context and culture, this kind of music represents an ecologically valid example of music that has been used to bring people together. This makes it a good candidate in the context of studying music-induced autonomic synchronization. Second, we had access to the 224-stops Tamburini organ of the Messina Cathedral. This instrument offers a broad range of tones and sounds, thus affording at the same time a rich listening experience and practical feasibility (e.g., only one performing musician and no external amplification is needed). The concert program was designed to include excerpts that differed in terms of both basic auditory features and subjective appreciation.

**Table 1:** Details of the music excerpts.

**Author**	**Title**	**Year**	**Abbreviation**
Simone Quaroni	Harmonic progression	2012	Harmo
Simone Quaroni	Canzona alla Gabrieli	2012	Gabr
Léon Boëllmann	“Toccata” from Suite gothique, Op. 25	1895	Tocc
Johann Sebastian Bach	“Toccata” in D minor, BWV 565	ca. 1750	Bach
Léon Boëllmann	“Prière” from Suite gothique, Op. 25	1895	Prière
William Monk	Abide with me (Eventide)	1861	Abide
Padre Davide da Bergamo	“Elevation” in D minor	ca. 1850	Elev

### Design

The two groups of participants were tested on two different days, and the recordings for each group were repeated twice, on two different days, for a total of 4 days of recordings. On each day, each excerpt was performed twice, summing to a total of eight recordings for each excerpt. To our knowledge, this was the first study to investigate interpersonal synchronization of physiological rhythms during passive music listening. Therefore, we tested the same music excerpts multiple times with the aim of obtaining more robust estimates of interpersonal synchronization than we would have by playing each excerpt only once. However, the choice of repeating the same excerpts multiple times posed a limit to the total number of excerpts we could test without losing the attention and the collaboration of the listeners. The order of the excerpts was randomized across different days and groups.

### Measures

Using a previously validated wireless unit designed and built in our lab (Codrons et al., 2014), we collected from each participant the electrocardiogram, respiratory excursion, and finger vasomotion (see Supplementary Materials for more detailed information). Continuous non-invasive blood pressure was also simultaneously recorded from three participants in each group by radial artery applanation tonometry.

Subjective ratings of (1) music pleasantness and (2) familiarity with the music excerpts were collected following the first performance of the first day, expressed on 1–5 Likert scales (1 = not at all; 5 = very much). In terms of basic auditory features, we focused on the envelope profile of the amplitude (loudness) of the music excerpt, as this dimension has been previously shown to faithfully entrain the spontaneous oscillations of cardiovascular autonomic variables (Bernardi et al., 2009). The music envelope was acquired simultaneously to the rest of the biological signals recorded from all the participants. The music envelope is a low-frequency signal proportional to the amplitude of the audio signal, and was obtained by feeding the music signal through an envelope generator (Electro Harmonix BIFilter, Long Island city, NY, USA). The music envelope was constructed by first inverting the negative part of the audio signal, then tracking the signal peaks as a continuous function of time. This part of the signal treatment was obtained with a flat frequency response from 40 Hz to 20 kHz. Finally, the envelope signal was low-pass filtered (cut-off 0.83 Hz, −20 dB/decade). Thus, the resulting envelope was insensitive to the changes in the frequency of the musical signal (high or low pitch), but only to the changes in its amplitude over time. For each music excerpt, we computed a loudness variability index, as the coefficient of variation of the envelope profile (standard deviation/mean ^*^ 100). Considering the lack of any previous study on the topic of music-induced interpersonal synchronization, we chose to focus on the music loudness variability as, to our knowledge, this is the only dimension that has been clearly shown in previous literature to promote instantaneous synchronism with the music, at least on an individual level (Bernardi et al., 2009).

A quantitative analysis of the degree of synchronization across individuals was done using the Generalized Partial Directed Coherence (Baccalá et al., 2007; see Supplementary Materials for more detailed information). This method provides direct structural information for multivariate autoregressive models that simultaneously model many time series, and is therefore well suited to capture the complex interactions among a group of individuals. In the coherence spectra, the reported values span between 0 and 1, where 0 is associated with total asynchrony and 1 with absolute synchronization, thus the greater the value the greater the coordination between a given pair of participants in that signal and during that recording. For each of the spectra we extracted the average coherence in the low-frequency band (0.035–0.15 Hz, LF) and in the high-frequency band (0.15–0.40 Hz, HF), as these bands are frequently assessed to test the autonomic modulation on heart rate variability (Bernardi et al., 2011).

### Statistical analyses

Statistical analyses were run using SPSS (version 23). Differences in synchronism due to different experimental conditions and groups were evaluated by multivariate analyses of variance (MANOVA). A separate MANOVA was run for each physiological signal. For the primary analysis of this study, we evaluated the effect of music as a whole on interpersonal autonomic synchronization, regardless of the specific music excerpt. To this end, we averaged the coherence scores between the seven music excerpts, thus yielding a single “music” coherence score for each pair of participants. The coherence scores related to listening to music for each pair of participants where compared to those obtained during the initial resting baseline. This was done in a series of bivariate two-way MANOVAs, one for each of the four physiological signals. The coherence scores for the LF and HF frequency bands were treated as dependent variables, whereas the experimental condition (music vs. baseline) and group membership (musicians vs. non-musicians) were treated as independent variables. The *p*-values resulting from these analyses were Bonferroni-corrected for multiple comparisons, taking into account 32 comparisons (4 physiological signals × 2 frequency bands × 2 groups of participants × 2 experimental conditions), thus setting the alpha level at 0.05/32 = 0.0016. In a secondary exploratory analysis, we compared each individual music excerpt with the baseline, to investigate whether different excerpts result in different degrees of synchronization. The statistical design was similar to the primary analysis, with the difference that there were 8 experimental conditions (7 music excerpts and the baseline) instead of 2. Owing to the exploratory nature of this analysis, the *p*-values resulting from this secondary analysis were only Bonferroni-corrected taking into account seven comparisons, i.e., the comparison of each excerpt with the baseline.

The loudness variability index, averaged across the eight performances of each excerpt, the ratings of pleasantness and the ratings of familiarity were used as independent variables in a stepwise linear regression analysis to identify reliable predictors of group autonomic synchronization (also averaged across the eight performances of each excerpt).

The degree of stability over multiple recordings of multivariate group synchronization measures is not known. To verify that changes in synchronization due to our experimental manipulations were not purely the by-product of test-retest variability, we run additional analyses to compare the coherence scores between the initial baseline conditions recorded on the 2 days of testing. Four bivariate one-way MANOVAs were run for this purpose, one for each of the four physiological signals, with the LF and HF coherence scores at baseline as the dependent variables, and the day of testing (day 1 vs. day 2) as the only independent variable. The *p*-values resulting from these analyses were Bonferroni-corrected for multiple comparisons, taking into account eight comparisons (4 physiological signals × 2 frequency bands).

## Results

### Primary analysis: music as a whole increases group synchronization

**Figure 1:**
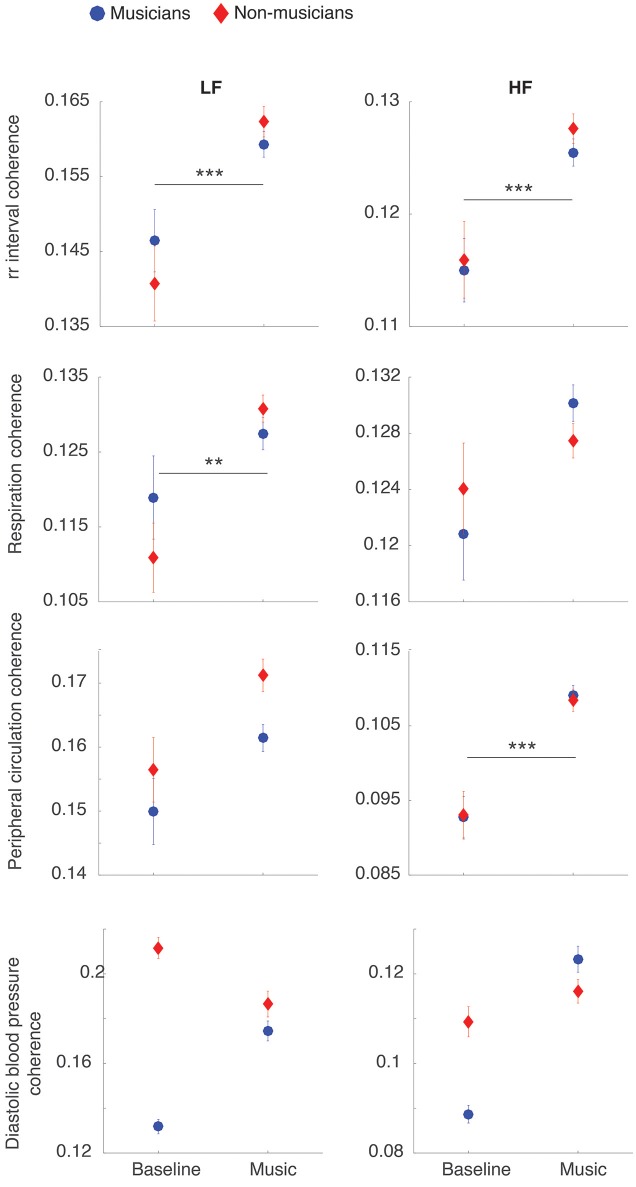
Group synchronization of physiological rhythms increases during passively listening to music. The figure shows the degree of interpersonal synchronization (mean ± SE of coherence scores) during the resting baseline and during passively listening to music. For the purpose of this analysis, the coherence scores from different music excerpts have been averaged together. It can be seen that music results in an increase of interpersonal coherence of heart rate, respiration and peripheral circulation, compared to baseline. ^**^p < 0.01; ^***^p < 0.001.

Figure 1 shows the degree of interpersonal group synchronization of physiological signals during passively listening to music. We observed a small but reliable increase in group synchronization during the music condition compared to baseline. This was statistically significant in the case of the RR interval [main effect of experimental condition, LF: F_(1, 1086)_ = 24.0, *p* < 0.001, η^2^p = 0.02; HF: F_(1, 1086)_ = 30.1, *p* < 0.001, η^2^p = 0.03; *p*-values corrected], respiratory excursion [main effect of experimental condition, LF: F_(1, 944)_ = 14.9, *p* = 0.004, η^2^p = 0.02; HF: F_(1, 944)_ = 6.5, *p* = 0.35, *n.s*.] and peripheral circulation [main effect of experimental condition, LF: F_(1, 914)_ = 7.8, *p* = 0.17, *n.s*.; HF: F_(1, 914)_ = 40.7, *p* < 0.001, η^2^p = 0.04]. A similar trend was also observed for blood pressure, but this was not statistically significant (LF and HF: both *p* > 0.8), likely due to the limited data points available for this variable. No statistically significant main effect of group or interaction of group with experimental condition was found following correction for multiple comparisons.

### Secondary exploratory analysis: effects of different music excerpts

**Figure 2:**
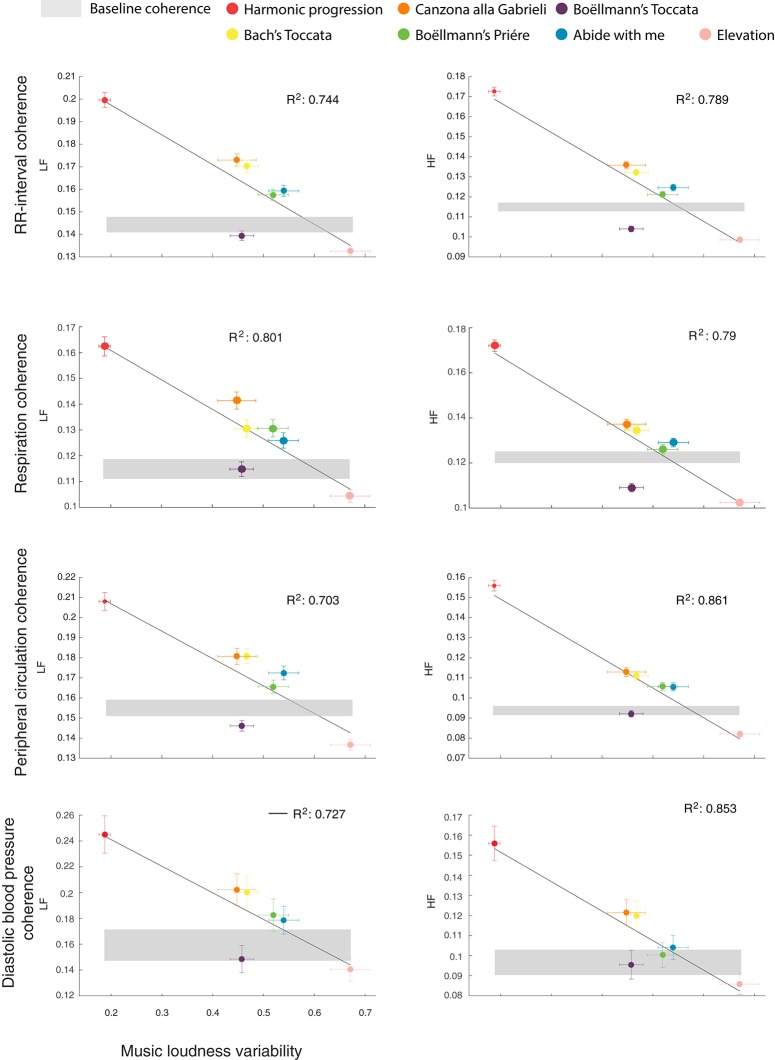
Changes in group synchronization heavily depend on low-level auditory feature. Each dot represents one of the seven music excerpts, plotted as a function of group synchronization (y axis), averaged across musicians and non-musicians, and loudness variability index (x axis). Vertical error bars represent variability in synchronization across different individuals and days. Horizontal error bars represent variability in the loudness variability index across different performances of the same excerpt. The shaded gray area represents the average group synchronization during the initial resting baseline (mean ± SE). Dots below and above the gray shaded area indicate decreased and increased group synchronization compared to baseline, respectively, (see Table 2 for a statistical account of the comparison with baseline). It can be seen that greater autonomic synchronization is achieved for music excerpts with lower loudness variability index.

Figure 2 shows the absolute level of synchronization, for each music excerpt and for each physiological signal, whereas Table 2 shows the relative changes in synchronization compared to baseline and the details of the statistical analyses. Maximum synchronization compared to baseline was achieved during listening to the Harmonic progression, for all physiological signals (*p* < 0.001). A lower degree of synchronization, but still significantly higher than baseline, was observed during Bach's “Toccata” and during the “Canzona alla Gabrieli” (*p* < 0.05 or better). Group synchronization remained for the most part at baseline level during the hymn “Abide with Me” and the Boëllman's “Prière.” Interestingly, certain excerpts appeared to actively disrupt group synchronization of physiological rhythms. In fact, we observed a significant decrease in synchronization compared to baseline during listening to the Boëllman's “Toccata,” and an even stronger de-synchronization during Padre Davide's “Elevation” (*p* < 0.05 or better). The pattern of autonomic synchronization was very similar between musicians and non-musicians. However, musicians showed overall stronger group synchronization, and weaker group de-synchronization, compared to non-musicians, in particular in their breathing and peripheral circulation (*p* < 0.01).

**Table 2:** Effect of different music excerpts on group synchronization of physiological signals.

			**Music excerpts**							**MANOVA**		
			**Harmo**	**Gabr**	**Tocc**	**Bach**	**Prière**	**Abide**	**Elev**	**Music excerpt**	**Group**	**Music excerpt by group**
**Mean loudness variability**			**0.19**	**0.45**	**0.46**	**0.47**	**0.52**	**0.54**	**0.67**			
RR interval	Musicians	LF	0.052	0.026	−0.008	0.024	0.012	0.013	−0.014	LF : F_(7, 7488)_ = 66	LF : F_(1, 7488)_ = 0.5	LF : F_(7, 7488)_ = 0.9
		HF	0.060	0.020	−0.009	0.017	0.008	0.012	−0.015	*p* < 0.001, η^2^p = 0.06	*p* : ns	*p* : ns
	Non-musicians	LF	0.060	0.033	−0.002	0.028	0.017	0.021	−0.011	HF : F_(7, 7488)_ = 184	HF : F_(1, 7488)_ = 0.1	HF : F_(7, 7488)_ = 1.0
		HF	0.059	0.021	−0.012	0.017	0.006	0.008	−0.018	*p* < 0.001, η^2^p = 0.15	*p* : ns	*p* : ns
Thoracic breathing	Musicians	LF	0.042	0.021	−0.005	0.012	0.009	0.008	−0.014	LF : F_(7, 6040)_ = 32	LF : F_(1, 6040)_ = 0.1	LF : F_(7, 6040)_ = 0.6
		HF	0.054	0.016	−0.012	0.015	0.004	0.007	−0.019	*p* < 0.001, η^2^p = 0.04	*p* : ns	*p* : ns
	Non-musicians	LF	0.054	0.031	0.004	0.018	0.020	0.014	−0.006	HF : F_(7, 6040)_ = 105	HF : F_(1, 6040)_ = 6	HF : F_(7, 6040)_ = 2
		HF	0.048	0.015	−0.017	0.009	0.000	0.005	−0.022	*p* < 0.001, η^2^p = 0.11	*p =* 0.009, η^2^p = 0.001	*p* = 0.038, η^2^p = 0.002
Peripheral	Musicians	LF	0.056	0.031	−0.005	0.028	0.016	0.023	−0.013	LF : F_(7, 5042)_ = 44	LF : F_(1, 5042)_ = 7	LF : F_(7, 5042)_ = 0.3
circulation		HF	0.066	0.021	0.000	0.021	0.016	0.015	−0.007	*p* < 0.001, η^2^p = 0.06	*p* = 0.009, η^2^p = 0.001	*p* : ns
	Non-musicians	LF	0.046	0.021	−0.011	0.020	0.006	0.014	−0.025	HF : F_(7, 5042)_ = 125	HF : F_(1, 5042)_ = 11	HF : F_(7, 5042)_ = 2
		HF	0.060	0.018	−0.005	0.014	0.010	0.010	−0.015	*p* < 0.001, η^2^p = 0.15	*p =* 0.001, η^2^p = 0.002	*p* = 0.032, η^2^p = 0.003
Diastolic blood	Musicians	LF	0.122	0.068	0.024	0.078	0.058	0.052	0.014	LF : F_(7, 426)_ = 7	LF : F_(1, 426)_ = 0.01	LF : F_(7, 426)_ = 1
pressure		HF	0.065	0.034	−0.002	0.030	0.009	0.012	−0.002	*p* < 0.001, η^2^p = 0.1	*p* : ns	*p* : ns
	Non-musicians	LF	0.030	0.011	−0.056	−0.029	−0.026	−0.027	−0.062	HF : F_(7, 426)_ = 8	HF : F_(1, 426)_ = 0.54	HF : F_(7, 426)_ = 0.9
		HF	0.048	0.017	−0.008	0.017	−0.002	−0.006	−0.017	*p* < 0.001, η^2^p = 0.13	*p* : ns	*p* : ns

*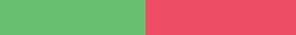 p < 0.001*.

*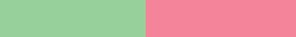 p < 0.01*.

*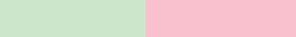 p < 0.05*.

*Cells show the amount of change in synchronization compared to baseline when participants listened to the different music excerpts. Positive values indicate an increase in synchronization (shown in green), negative values indicate a decrease in synchronization (shown in red). Color gradients represent the level of statistical significance of the post-hoc comparison with baseline synchronization (independent samples t-test with Bonferroni correction for multiple comparisons). The excerpts are ordered in terms of increasing auditory complexity, as evidenced by the loudness variability index, first row. The MANOVA columns show the F statistic, degrees of freedom, p value and effect size (Partial Eta Squared) for the main effects and interactions of the factors Music excerpt and Group membership p. LF, low frequencies; HF, high frequencies; Harmo, Harmonic progression; Gabr, Canzona alla Gabrieli; Tocc, Boëllmann's Toccata; Bach, Bach's D minor Toccata; Prière, Boëllmann's Prière; Abide, Abide with me; Elev, Padre Davide's Elevation*.

**Figure 3:**
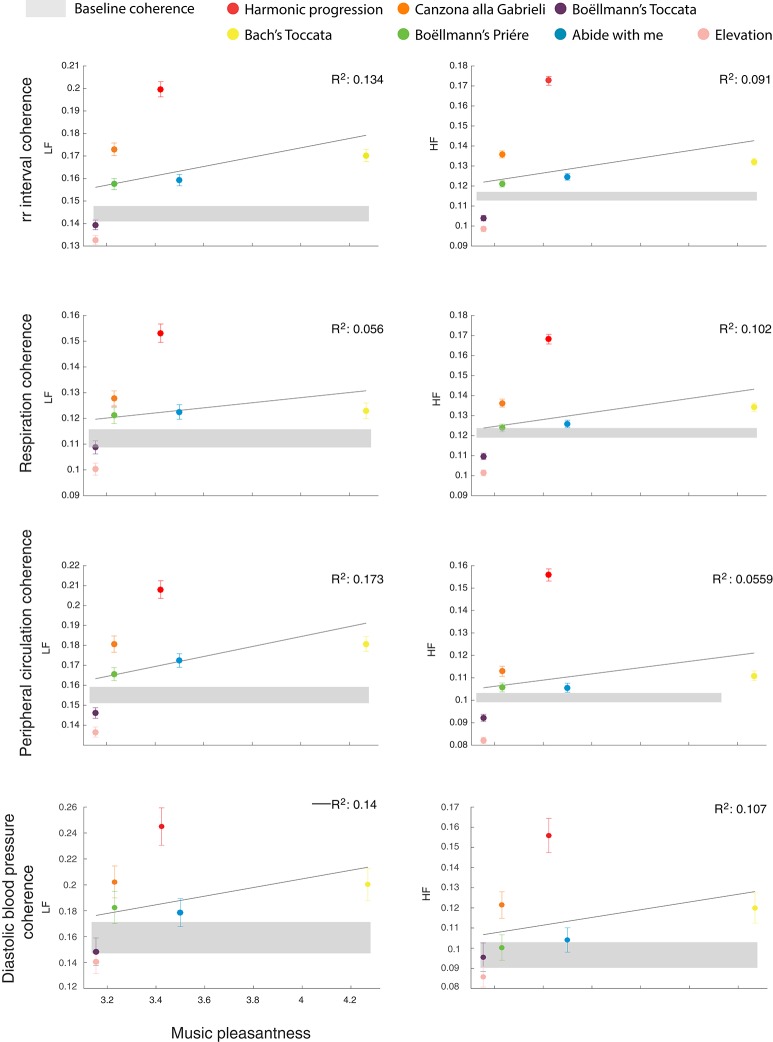
Changes in group synchronization show weak dependence on subjective music appreciation. Conventions are the same as in Figure 2, but here group synchronization is plotted as a function of subjective ratings of music pleasantness. A trend can be noticed toward greater group synchronization for music that is perceived as more pleasant, but the amount of variance in synchronization explained by this factor is low.

These results show that physiological signals may synchronize between individuals during listening to music, but also that this is not always the case. Whereas, some excerpts induce robust and widespread synchronization, other music may trigger highly idiosyncratic physiological patterns, decreasing synchronization below baseline levels. We then searched for factors that could explain the diverse effects on synchronization of the various music excerpts. We focused on two highly distinct dimensions, both of which are known to play an important role in determining the response to music: subjective appreciation, subdivided in pleasantness and familiarity, and loudness variability. As shown in Figure 2, excerpts with simpler (i.e., less variable) loudness structure resulted in greater group synchronization, whereas music excerpts with more complex auditory pattern result in decreased group synchronization. Accordingly, for all physiological signals, stepwise regression analyses indicated loudness variability as the only reliable predictor of group synchronization, explaining alone up to 80% of the variance in group synchronization (all *p* < 0.01, all *R*^2^ > 0.7; the correlation between loudness variability and the various physiological signals was still observable after removing from the sample the music excerpt Harmonic progression, yielding a mean Pearson correlation coefficient of *r*_(6)_ = −0.67 ± 0.04, averaged across the 2 frequency bands and the 4 physiological signals). Figure 3 shows the correlation between group synchronization and the ratings of pleasantness (the ratings of familiarity yielded highly similar results, and therefore the data are not shown). Subjective ratings of pleasantness or familiarity explained a smaller portion of the variance in group synchronization (all *R*^2^ < 0.2), with excerpts rated as more pleasant or more familiar yielding greater synchronization. Possibly, subjective appreciation drove synchronization when comparing excerpts with similar loudness variability. This can be seen in the Bach “Toccata,” which was rated as highly appreciated and yielded stronger synchronization compared to other excerpts with similar auditory complexity. Similarly, subjective appreciation possibly accounted for the fact that listening to Boëllman's “Toccata” resulted in a significant decrease in synchronization compared to baseline, although it had similar loudness variability index to Bach's “Toccata.”

### Stability of the coherence measures

No statistically significant changes in the coherence scores at baseline were found between the first and the second day of testing, following correction for multiple comparisons, for any of the four physiological signals investigated (*p* > 0.27 or higher). This suggests that the changes in coherence observed as a result of listening to music were not due to random variability occurring as a result of repeating the measurements several times.

## Discussion

We showed that passively listening to music increases interpersonal synchronization of cardiovascular and respiratory rhythms. It has been suggested that one of the functions that music may serve is binding social groups together (Fitch, 2006). Accordingly, the social dimension has been recently identified as one of the statistical quasi-universals characterizing human music productions across the globe (Savage et al., 2015). The present study provides a mechanistic explanation of how social bonds may be created during exposure to music, namely through the synchronization of physiological rhythms. Music stimulation is capable of influencing the autonomic nervous system both directly, through the convergence of auditory and vegetative pathways at the level of the reticular formation (Cant and Benson, 2003) and indirectly, through various cortical and subcortical pathways (Koelsch, 2010). A growing body of literature points to the synchronization of physiological rhythms between individuals as a marker of emotional bonding (see Palumbo et al., 2016, for a review). Interpersonal autonomic synchronization has been found to be greater, for example, between individuals within a romantic relationship (Helm et al., 2012), in psychotherapists who are more emphatic to their clients (Robinson et al., 1982), and in game players who report enhanced sense of “being in the zone” with each other (Noy et al., 2014).

Importantly, our findings also highlight that not *all music* promotes interpersonal autonomic synchronization. A strong relationship was found between the complexity of the loudness profile of the music and the degree of interpersonal synchronization. Music with a simpler structure resulted in greater autonomic group synchronization, whereas music with more complex loudness profile was shown to disrupt interpersonal synchronism even below baseline level. It is well established that different music may evoke different and even opposite physiological responses. For example, it has been shown that music may increase or decrease sympathetic arousal depending on the music tempi (Bernardi et al., 2006). Along a similar line, and relevant for the current investigation, we have previously shown that the synchronization between an individual's finger vasomotion and the music can be relatively stronger or weaker depending on the structure of the music loudness profile (Bernardi et al., 2009). In continuity with these previous findings, here we show that music may increase or decrease interpersonal synchronization, depending on the variability of the music loudness profile. Our findings are consistent with previous investigations showing that simpler auditory templates result in maximal group motor synchronization (Codrons et al., 2014). These previous as well as the current findings reveal that the simplest music has the most relevant synchronizing effects, or in other words, it creates the more “consensus.” This concept might have significant practical implications for the development of soundtracks to improve bonding between individuals that need to perform a common task.

Subjective liking or familiarity with the music excerpts seemed to play a relatively minor role in explaining interpersonal synchronization. This observation mirrors the results of several previous investigations showing that the contribution of personal preferences to the physiological response to music is limited, compared to the auditory features of the musical stimuli (Iwanaga and Moroki, 1999; Nater et al., 2005, 2006; Bernardi et al., 2006, 2009; Pérez-Lloret et al., 2014; Vlachopoulos et al., 2015). On the other, our findings provide preliminary evidence for the novel hypothesis that greater subjective appreciation might increase interpersonal synchronization when comparing music excerpts with similar basic auditory features.

Our findings also suggest that previous music training is not a crucial factor in the emergence of interpersonal autonomic synchronization during music listening. In fact, musicians and non-musicians showed the same overall pattern of responses, with increased synchronization for simpler music and decreased synchronization when listening to the more complex excerpts. These findings point to the interesting fact that the implicit knowledge of music of non-trained individuals (Attneave and Olson, 1971; Dewar et al., 1977; Krumhansl and Shepard, 1979) is sufficient for interpersonal synchronization of physiological rhythms to emerge. On the other hand, in both sets of excerpts musicians showed a consistent pattern toward greater synchronization (or weaker de-synchronization for the more complex excerpts). Such increased synchronization is consistent with previous observations showing that musicians have a more pronounced reactivity to music stimuli, such as, a greater increase in respiratory rate during music listening (Bernardi et al., 2006).

It should be noted that the absolute level of synchronization we observed during music listening seems rather low (coherence < 0.2), and also that the extent of the increase in synchronization compared to baseline is small (+ ~0.05 coherence at most). On the one hand, the multivariate method we used generates estimates of coherence that are lower than those resulting from a more traditional bivariate approach, from which common norms to assess synchronization are derived (e.g., strong synchronization for coherence >0.8). The latter, however, reduces the group to a series of pairs and therefore is inappropriate to model a group of individuals as a dynamical system. Furthermore, it should be noted that the participants in our experiment were not given an explicit task beyond passively listening, they did not share common goals, nor they engaged in any motoric action. It is unlikely to observe large changes in autonomic synchronization under these conditions, and the small but robust changes described here are likely to become amplified in a more naturalistic situation where individuals would engage more actively.

A limitation of the present study lies in the small number of music excerpts tested (*n* = 7), and in the fact that all the excerpts belonged to the classical religious or religious-like organ repertoire. This limitation is of particular importance when assessing the correlation between the loudness variability and autonomic group synchronization, which should be treated as exploratory given the limited number of data points. Furthermore, the music excerpts we selected were focused on Christianity, which was the confession of the majority of participants. Future research employing a larger and more diverse corpus of excerpts, as well as more heterogeneous groups of study participants, is needed to confirm the observations made here and generalize the results to other kinds of music and populations. Another limitation is the relatively small number of groups tested (two groups, with 13–14 participants each), which limits the statistical power of our study. On the other hand, the multimodal physiological characterization of interpersonal synchronism we provided shows converging results from four different and partly independent physiological measures that together describe a robust and coherent pattern. These results will be ideally complemented by future studies applying a more basic physiological monitoring, such as, that available through consumer mobile technologies, to a very large number of participants simultaneously. Furthermore, in this study we only took loudness variability into account in terms of music structural features. It will be interesting in future studies to address to role of other dimensions such as, the rhythm, meter, melody, and emotional valence of the music excerpts.

To conclude, we shall notice that simple rhythms and melodies largely dominate the choice of music during rituals and mass events, situations in which the value of group cohesion is highlighted. Our findings suggest that this choice may be based on the fact that it is precisely this kind of music that has the maximum potential to synchronize bodily rhythms across individuals, hence creating the biological soil for an elevated sense of togetherness.

## Author contributions

NB, EC, and LB: designed the study. EC, RdL, MV, FC, GV, and LB: collected the data. NB and LB: contributed analytic tools. NB and EC: analyzed the data. NB: drafted the manuscript. EC, RdL, MV, FC, GV, and LB: revised the draft paper.

### Conflict of interest statement

The authors declare that the research was conducted in the absence of any commercial or financial relationships that could be construed as a potential conflict of interest. The reviewer AC and handling Editor declared their shared affiliation.
