# Supplementary material for: Increase in Synchronization of Autonomic Rhythms between Individuals When Listening to Music
Source: Front Physiol. 2017 Oct 17;8:785. doi: 10.3389/fphys.2017.00785 (PMC5651050; doi:10.3389/fphys.2017.00785)
Supplement: Supplementary file 1 [file DataSheet1.doc]

**Title**

Increase in synchronization of autonomic rhythms between individuals when listening to music

**Authors**

Nicolò F. Bernardi^1,2^, Erwan Codrons^3^, Rita di Leo^4^, Matteo Vandoni^3^, Filippo Cavallaro^5^, Giuseppe Vita^5^, Luciano Bernardi^6^

**Affiliations**

^1^International Laboratory for Brain, Music and Sound Research, Montréal, Québec, Canada

^2^Department of Psychology, McGill University, Montréal, Québec, Canada

^3^Department of Public Health and Neuroscience, University of Pavia, Italy

^4^Neurology Unit, San Giovanni e Paolo Hospital, Venice, Italy

^5^Neurology Unit, Department of Clinical and Experimental Medicine, University of Messina, Italy

^6^Folkälsan Institute of Genetics, Folkälsan Research Center, University of Helsinki, Finland

**Supplementary methods**

*Equipment*

Measurements from each participant were obtained through a special microprocessor-driven portable unit.^1^ Participants wore their unit clipped to their trousers. This unit was capable to obtain one electrocardiogram from 3 standard thoracic leads (to obtain a bipolar D2 derivation, in order to record a well defined positive R wave), respiratory excursions from the abdomen and from the chest using the technique of inductive plethysmography (which consisted in positioning 2 highly flexible elastic belts around the upper chest and the abdomen just below the xyfoid), and finger vasomotion using a photoplethysmograph. Each unit included a 12-bit data acquisition system which digitized the data at the frequency of 400Hz/channel on a total of 12 channels.

In order to obtain synchronisation of all the recordings at the same time, each unit was equipped with an XBEE (Digi International Inc., Minnetonka, MN, USA) radio module (more information about XBEE units and about how to obtain a network of units can be found at: [http://www.digi.com/products/wireless-wired-embedded-solutions/zigbee-rf-modules/point-multipoint-rfmodules/xbee-series1-module#overview](http://www.digi.com/products/wireless-wired-embedded-solutions/zigbee-rf-modules/point-multipoint-rfmodules/xbee-series1-module#overview), and: <http://www.digi.com/pdf/ds_xbeemultipointmodules.pdf>). One additional radio module was connected to a Macintosh (MacBook Air) portable laptop which served as “coordinator”. This constituted a wireless network that could perform several operations on either individual units (e.g., checking signal quality, adjusting the gain on one signal if needed), by linking the laptop-coordinator to one specific external module or to all units simultaneously. Each unit stored the data on a Secure Digital memory card of 2GB memory as binary files. A specific code automatically named each file in order to easily identify each participant and each recording. Accordingly, recordings could be started and stopped from the laptop to all units simultaneously, hence providing synchronised files with corresponding file names. Once the acquisition was completed, data were uploaded on the computer and evaluated. Tests on synchronisation showed that the actual time difference in a 4 minute recording was below 20ms across all units.

During the entire experiment the music was continuously recorded on a H4n digital stereo recorder (Zoom corp., Tokyo, Japan) at the sampling rate of 96Khz/24 bit precision, and the music data were stored on 4GB SD cards. In order to synchronise the music with the biological signals during each recording we obtained on-line a low-frequency signal proportional to the amplitude of the audio signal. For this purpose, we derived an audio signal from the “phone” output of the recorder, and fed it to an envelope generator (Electro Harmonix BI- Filter, Long Island city, NY, USA). The music envelope was constructed by first inverting the negative part of the audio signal, then tracking the signal peaks as a continuous function of time. This part of the signal treatment was obtained with a flat frequency response from 40Hz to 20kHz. Finally, the envelope signal was low-pass filtered (cut-off 0.83 Hz, -20dB/decade). Thus, the resulting envelope was insensitive to the changes in the frequency of the musical signal (high or low pitch), but only to the changes in its amplitude over time. This low-frequency signal could be recorded using a similar portable unit as those applied to the subjects, and synchronised with the other via the same wireless system. Using this method, the music envelope could be acquired simultaneously to the rest of the biological signals recorded from all the participants.

*Data analysis*

Signal pre-analysis

We first obtained the heart period sequence from the electrocardiogram. This was done by first identifying the peak of the R wave of the ECG in the electrocardiogram, and then constructing the series of the heart period by measuring the R-R interval. This sequence of R-R intervals was converted into a continuous signal at a frequency of 4 Hz by interpolation of the R-R intervals at each data point. All the other signals (thoracic and abdominal respiration, finger vasomotion) were also directly re-sampled to 4 Hz. Before mathematical analysis, the data underwent linear de-trending of the signals to remove possible baseline drifts of signals.

Multivariate analysis of individual participants

A quantitative analysis of the degree of synchronisation was done with a multivariate coherence method. In recent years a series of frequency domain approaches were described to assess the relationship (intensity and direction of information flow) between multivariate time series, based on the decomposition of multivariate partial coherences computed from multivariate autoregressive models. New algorithms, called Partial Directed Coherence and Generalized Partial Directed Coherence (GPDC)^2^ provide direct structural information for multivariate autoregressive models that simultaneously model many time series.^3^ GPDC is considered an improvement of Partial Directed Coherence as it corrected some of its inaccuracies^4^ and for this reason it was used in the present study. GPDC is used to find the existence of direct connections between pairs of data sets, but in addition to a simple bi-variate model GPDC includes in the calculation the influence of the n-2 remaining sets. Thus, although GPDC would provide a matrix showing in each panel the coherences between pairs of signals (similar to a simpler bi-variate coherence), it does now in the context of a multivariate model, as GPDC considers all participants simultaneously. Furthermore, GPDC decomposes the interaction of the whole data series into directional components (forward and backward influences). The GPDC and derived methods are based on the concept of Granger causality, which states that if some time series Y(t) contains information in past terms that helps in the prediction of another time series X(t), then Y(t) is said to cause X(t).^5^ Further technical details and rationale for its use can be found in ^2^. This method was implemented from the Matlab routines MVAR and MVFREQZ (^6^, available for download at: http://biosig.sourceforge.net/index.html). We first constructed for each recording the multivariate data matrix, an array containing the sequences of one signal for each of the 10 subjects. This array was used for the autoregressive Partial Correlation Estimation, with the model order set to 12, the frequency range set to 0-2 Hz and the number of frequencies set to 400 (thus, setting the frequency resolution to 0.005 Hz). The autoregressive Partial Correlation Estimation was obtained by selecting the Nutall-Strand unbiased correlation function.^7^ The multivariate autoregressive model parameters were than used to compute GPDC. The generalized partial directed coherence in the frequency domain *f* was defined as:^2^

where σ and σ^2^ are the standard deviation and variance of the innovation processes *w,* respectively*, A_kj_ (f)* are the autoregressive terms of pairs k and j of time series (spanning from 1 to N data) and * denotes complex conjugate.

Using the data resampled which were obtained in each group of 10 participants and in each of the different conditions of the experiment and for each signal, and applying the GPDC method, we obtained 10x10 matrices of coherence spectra. Supplementary Figure 1 shows an example, related to the RR interval signal, obtained during listening to the Harmonic progression in the non-musicians group on the first day of recording. In the coherence spectra, the reported values span between 0 and 1, where 0 is associated with total asynchrony and 1 with absolute synchronisation, thus the greater the value the greater the coordination between the participants in that signal and during that recording. For each of the spectra obtained in each matrix, (except the comparison of each participant with himself), we extracted the average coherence in the low-frequency band (0.035 to 0.15Hz, LF) and in the high-frequency band (0.15 to 0.40Hz, HF). These coherence values (obtained from each of the pairs in the matrix except for the diagonal) were used for statistical analysis. The LF and HF band are frequently assessed to test the autonomic modulation on heart rate variability.^8^

**References**

1. Codrons E, Bernardi NF, Vandoni M, Bernardi L. Spontaneous group synchronization of movements and respiratory rhythms. PLoS One. 2014 Sep 12;9(9):e107538.
2. Baccalá LA, Sameshima K, Takahashi DY. Generalized partial directed coherence. In 15th International Conference on Digital Signal Processing 2007 Jul 1 (pp. 163-166). IEEE.
3. Baccalá LA, Sameshima K. Partial directed coherence: a new concept in neural structure determination. Biological cybernetics. 2001 May 1;84(6):463-74.
4. Schelter B, Timmer J, Eichler M. Assessing the strength of directed influences among neural signals using renormalized partial directed coherence. Journal of neuroscience methods. 2009 Apr 30;179(1):121-30.
5. Blinowska KJ. Review of the methods of determination of directed connectivity from multichannel data. Medical & biological engineering & computing. 2011 May 1;49(5):521-9.
6. Schlögl A. A comparison of multivariate autoregressive estimators. Signal processing. 2006 Sep 30;86(9):2426-9.
7. Marple Jr SL. Digital spectral analysis with applications. Englewood Cliffs, NJ, Prentice-Hall, Inc., 1987.
8. Bernardi L, et al. Methods of investigation for cardiac autonomic dysfunction in human research studies. Diabetes/metabolism research and reviews. 2011 Oct 1;27(7):654-64.

*Supplementary Table 1*

Participants’ information

|  | Age | MBI | Music instrument | Age start training | Smoke | Sport |
| --- | --- | --- | --- | --- | --- | --- |
| Non-musicians |
| 01 | 31 | 22.5 | n.a. | n.a. | 4 | Soccer, 2*1 |
| 02 | 53 | 20.8 | n.a. | n.a. | 0 | 0 |
| 03 | 26 | 25.7 | n.a. | n.a. | 0 | Tennis, 3*1.5 |
| 04 | 27 | 31.9 | n.a. | n.a. | 70 | 0 |
| 05 | 47 | 22.0 | n.a. | n.a. | 150 | 0 |
| 06 | 57 | 25.6 | n.a. | n.a. | 0 | 0 |
| 07 | 31 | 26.6 | n.a. | n.a. | 140 | 0 |
| 08 | 45 | 27.0 | n.a. | n.a. | 200 | 0 |
| 09 | 53 | 31.2 | n.a. | n.a. | 0 | 0 |
| 10 | 51 | 27.2 | n.a. | n.a. | 0 | 0 |
| 11 | 27 | 23.8 | n.a. | n.a. | 100 | Gym, 3*1.5 |
| 12 | 31 | 20.8 | n.a. | n.a. | 10 | Aerobics 5*1.5 |
| 13 | 37 | 23.6 | n.a. | n.a. | 0 | 0 |
| Musicians |  |  |  |  |  |  |
| 01 | 31 | 21.2 | Guitar | 21 | 0 | 0 |
| 02 | 34 | 32.1 | Voice, piano | 3 | 0 | Cardiofit, 2*2 |
| 03 | 53 | 21.5 | Voice | 45 | 50 | 0 |
| 04 | 54 | 24.4 | Voice | 13 | 0 | 0 |
| 05 | 47 | 22.7 | Voice | 30 | 0 | 0 |
| 06 | 53 | 21.3 | Voice | 30 | 0 | Dance, 2*1 |
| 07 | 26 | 19.6 | Guitar | 16 | 0 | 0 |
| 08 | 44 | 26.6 | Voice | 12 | 0 | 0 |
| 09 | 63 | 27.6 | Voice | 26 | 3 | 0 |
| 10 | 65 | 25.7 | Voice | 11 | 0 | Bike, 5*0.5 |
| 11 | 54 | 21.1 | Voice | 15 | 0 | Jogging, 2*1 |
| 12 | 32 | 23.7 | Voice | 19 | 0 | 0 |
| 13 | 53 | 22.7 | Percussions | 17 | 0 | 0 |
| 14 | 62 | 34.5 | Voice | 10 | 0 | 0 |

*Supplementary Figure 1*

Generalized Partial Directed Coherence (GPDC)


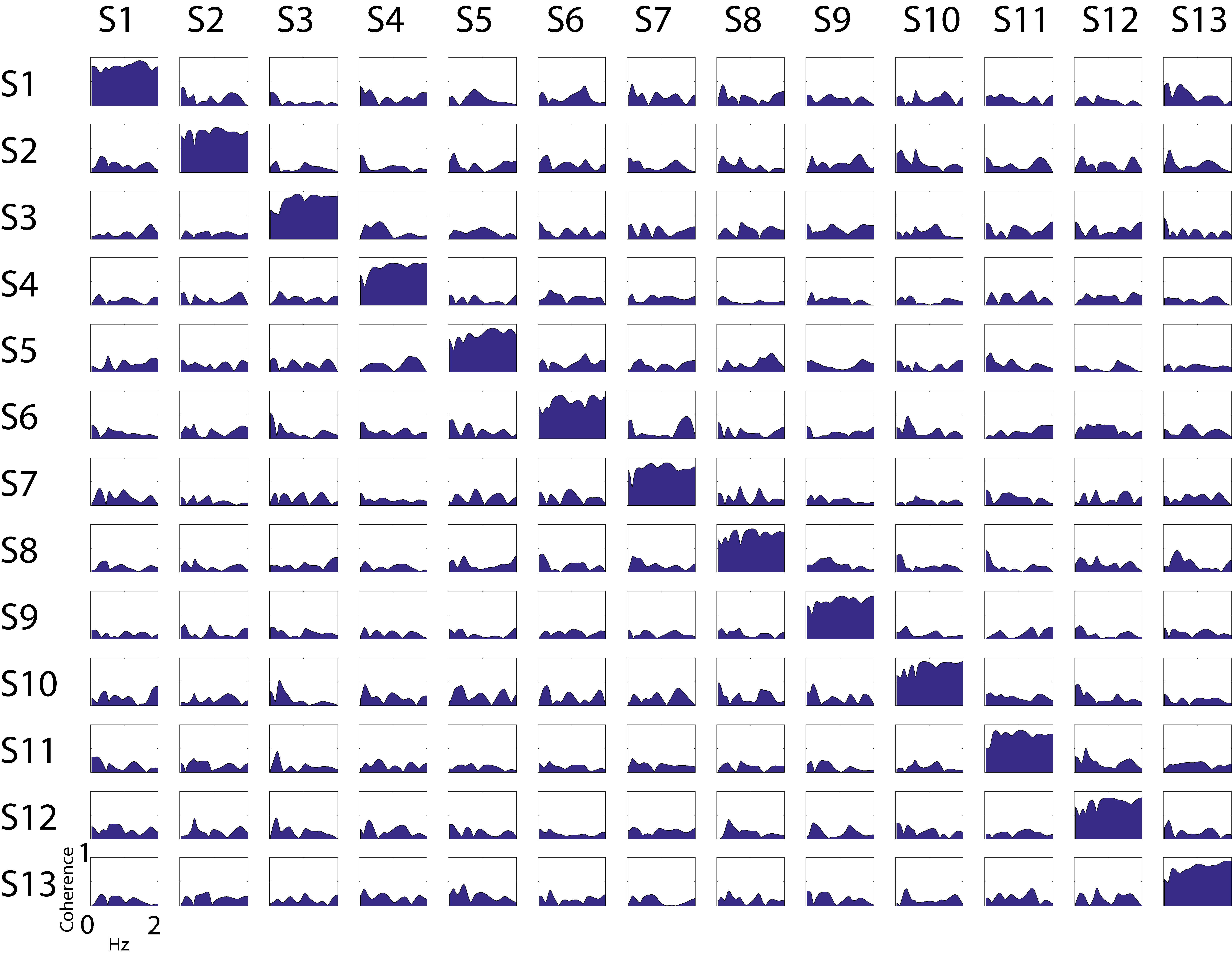


Example of multivariate (GPDC) coherence spectra matrix, related to the RR interval signal, obtained during listening to the Harmonic progression in the 13 non-musicians (S1–S13) on the first day of recording. In each spectrum the abscissa reports the frequency in Hz, and the ordinate reports the coherence, from 0 to 1. Each subject is compared with all the other subjects and with him/herself in the diagonal. Note that the coherence in the diagonal is lower than 1, as each subject is compared with himself, but in the context of a multivariate model that takes into account also the remaining subjects.
